# Differentiation between physical and chemical effects of oil presence in freshly spiked soil during rhizoremediation trial

**DOI:** 10.1007/s11356-019-04819-6

**Published:** 2019-05-02

**Authors:** Imran Hussain, Markus Puschenreiter, Soja Gerhard, Syed Gul Abbas Shah Sani, Waqas-us-din Khan, Thomas G. Reichenauer

**Affiliations:** 1 AIT Austrian Institute of Technology, Centre for Energy, Environmental Resources and Technologies, Tulln, Austria; 2 Department of Molecular Systems Biology, Faculty of Life sciences, University of Vienna, Vienna, Austria; 3 Department of Natural Resources and Environmental Engineering, Bioenergy and Environmental Remediation Lab (BERL), Hanyang, South Korea; 4 Department of Forest and Soil Sciences, University of Natural Resources and Life Sciences Vienna, Konrad Lorenz Straße 24, A-3430, Tulln, Austria; 5 Department of Plant Pathology, University of California Davis, Davis, CA USA; 6 Sustainable Development Study Centre, Government College University, Lahore, Pakistan

**Keywords:** Physical effects, Chemical toxicity, Diesel fuel, Vegetable oil, Plant growth, Rhizoremediation

## Abstract

Petroleum contamination and its remediation via plant-based solutions have got increasing attention by environmental scientists and engineers. In the current study, the physiological and growth responses of two diesel-tolerant plant species (tolerance limit: 1500–2000 mg/kg), Italian ryegrass (*Lolium multiflorum*) and Birdsfoot trefoil (*Lotus corniculatus*), have been investigated in vegetable oil– and diesel oil–amended soils. A long-term (147-day) greenhouse pot experiment was conducted to differentiate the main focus of the study: physical and chemical effects of oil (vegetable and diesel) in freshly spiked soils via evaluating the plant performance and hydrocarbon degradation. Moreover, plant performance was evaluated in terms of seed germination, plant shoot biomass, physiological parameters, and root biomass. Addition of both diesel oil and vegetable oil in freshly spiked soils showed deleterious effects on seedling emergence, root/shoot biomass, and chlorophyll content of grass and legume plants. Italian ryegrass showed more sensitivity in terms of germination rate to both vegetable and diesel oil as compared to non-contaminated soils while Birdsfoot trefoil reduced the germination rate only in diesel oil–impacted soils. The results of the current study suggest that both physical and chemical effects of oil pose negative effects of plant growth and root development. This observation may explain the phenomenon of reduced plant growth in aged/weathered contaminated soils during rhizoremediation experiments.

## Introduction

Organic contaminants like aliphatic, aromatic, heterocyclic and asphaltene/tar hydrocarbons are collectively termed as total petroleum hydrocarbons (TPHs) (Van Epps 2006). TPHs are an important category of environmental contaminants present in most of the agricultural and industrial countries throughout the world (Kamath et al. 2004; Huang et al. 2005). TPHs can enter the environment in the processes of exploration, extraction, and refining as well as via accidental oil spills and leakage of underground storage tanks (Aisien et al. 2015). Human health and ecosystem safety are directly influenced by accumulation of petroleum products in the environment (Tang et al. 2010). Prominent deleterious effects caused by TPH pollution are the destruction of soil structure, damages to water quality and inhibition of plant growth (Cunningham et al. 1996; Frick et al. 1999; Meagher 2000; Hutchinson et al. 2001; Besalatpour et al. 2008; Murakami et al. 2008; Jain et al. 2011). Thus, the eco-toxicity of TPHs provides motivation for environmental scientist and engineers for finding sustainable methods for the remediation of these compounds. A set of physical, chemical, biological and integrated approaches are available to deal with the problem of TPH-impacted sites (Khan et al. 2013a; Abhilash et al. 2009; Lim et al. 2016). Physical and chemical methods can offer quick and fast solutions for decontamination, but they require extensive input of material, heavy tools and labour (Broman et al. 1990; Khan et al. 2013b; Lim et al. 2016). Additional disadvantages linked with these methods are destruction of soil structure and consequently impairment of soil quality. They can be exhaustive to the environment and often have poor public acceptability (Lim et al. 2016). Phytoremediation—using plants for remediation of contaminated sites—is a sub-group of bioremediation (Gaskin and Bentham 2010; Gerhardt et al. 2009). Under certain circumstances, plants have the potential to enhance pollutant decontamination as compared to microbes alone (Bastida et al. 2016). Petroleum-contaminated soils can be treated by rhizoremediation also called rhizodegradation (Gerhardt et al. 2009; Ijaz et al. 2016a) which is based on the stimulation of hydrocarbon degrading microbial population in the rhizosphere of plant roots (i.e. 1–2 mm around plant roots) (Pichtel and Liskanen 2001; Qixing et al. 2011). Rhizodegradation can offer a viable option as an efficient and cost-effective remediation measure of petroleum-contaminated soils (Kuppusamy et al. 2015; Lim et al. 2016). A number of recent studies depicted the successful application of rhizoremediation for decontamination of TPH-contaminated soils (Al-Baldawi et al. 2015; Arslan et al. 2014a; Bramley-Alves et al. 2014; Cao et al. 2016; Dadrasnia and Agamuthu 2014; Fatima et al. 2015; Khatisashvili et al. 2015; Zhang et al. 2016). Rhizoremediation rate and performance can be stimulated by synergistic use of suitable microbes and plants (Chaudhry et al. 2005; Ijaz et al. 2016b; Lladó et al. 2013). Use of mixed plant species (grasses and legumes) can enhance the degradation rate due to a deeper root penetration and thus improved the stimulation of microbial communities (Phillips et al. 2009). The cultivation of mixed plant types is also believed to allow stimulation of rhizospheric effects in a more pronounced way via greater infiltration and stimulation of microbial biomass in the rhizosphere region. The underlying hypothesis is that mixed plant cultivation will add positive effects via summing the benefits of each individual plant during rhizoremediation trials (Phillips et al. 2009). A mixture of Italian ryegrass (IR) and Birdsfoot trefoil (BT) has been shown to be effective in degrading TPHs as both plant species are tolerant to TPH contamination and provide additional benefits such as the extensive fibrous root system of the grass and the potential supply of nitrogen by fixing it from the atmosphere by the legume (Fan et al. 2008; He et al. 2005; Kaimi et al. 2006; Phillips et al. 2009; Rezek et al. 2008; Smith et al. 2006). The presence of plants in hydrocarbon-contaminated soils can enhance the diversity, density and activity of TPH-degrading microbes by the action of root exudates (Baetz and Martinoia 2014). The degradation rate can be further accelerated by adding suitable organic (compost, manure, biochar) and inorganic (nutrients and fertiliser) soil amendments (Arslan et al. 2014a; Romantschuk et al. 2000). Until now, this technique has been shown with positive results in laboratory (Agostini et al. 2013; Beesley et al. 2011; Gerhardt et al. 2009), greenhouse (Euliss et al. 2008; Olson et al. 2007; Peng et al. 2009) and field-scale studies (Chien 2012; Phillips et al. 2009; Pizarro-Tobías et al. 2015) for a variety of soil types. Considering rhizoremediation of TPH-contaminated soils, root development is the most important factor that can affect microbial drift (change in microbial community structure as well as abundance of TPH-degrading microbes) in the rhizosphere, considered as a potential driving force for stimulated rhizoremediation (Baetz and Martinoia 2014; LeFevre et al. 2013; Martin et al. 2014). The significant increase in microbial biomass in the rhizosphere is a function of plant roots as it provides substrate and water for microbial growth and metabolisms (Merkl et al. 2004; Inckot et al. 2011; Smith et al. 2006; Shirdam et al. 2008; Hinsinger et al. 2005). In most of the rhizoremediation or phytoremediation studies (especially focused on TPH), researchers mainly evaluated the phytotoxicity to different plant species by the chemical effects of diesel- or petroleum-derived compounds. Here, chemical effects are referred to as direct toxic effects of persistent long-range compounds of diesel as well as its derived products and chemical changes in soil properties. Compounds especially long-chain alkanes (C13-C40) (Choi and Lee 2013) and aromatics of petroleum origin can cause deleterious effects on seedling emergence and plant growth parameters (Inckot et al. 2011; Olson et al. 2007; Smith et al. 2006). Less is reported about the physical effect of oil on plant growth (Gartler et al. 2014). Potential physical and chemical effects caused by the presence of oils in soil are less oxygen supply, destructed soil structure and poor nutrient exchange. The other reported physical effects of oil’s presence in soils are the decrease in soil temperature and water holding capacity as well as the increase in soil pH that may linked with lower phosphorus availability (Wang et al. 2017; Sonnleitner et al. 2003). Many rhizoremediation investigations showed that weathered or aged contaminated soils showed less phytotoxic effects as compared to freshly spiked soils which yielded more plant biomass. Nevertheless, some case studies also showed less shoot and root biomass in aged or weathered contaminated soils leading to poor degradation of TPH as compared to freshly spiked soil (Soleimani et al. 2010). This may be explained by the presence of physical and chemical effects caused by the presence of oil in soils. Additionally, in one of our experiments (Hussain et al., unpublished), mixed plants (Italian ryegrass and Birdsfoot trefoil) gave a significantly higher yield (5–10 times) in freshly spiked soils compared to aged contaminated soils having the same contamination load. Short-chained hydrocarbons are—due to their toxicity—made responsible for the negative chemical effects on plant performance, but their abundance is normally less in aged contaminated soils. The working hypothesis can be more elaborated as follows: the effects of diesel fuel on plants are caused by both chemical effects (e.g. small toxic substances) and physical effects (e.g. clogging of soil pores, reduction in water holding capacity of the soil). Thus, we compared soil amended with diesel fuel with soil amended with vegetable oil, which has similar physical properties (e.g. viscosity, density, hydrophobicity), but has mainly physical effects on soil and no direct toxic effects due to the lack of low-molecular-weight substances. Secondly, the aim of using vegetable oil was to simulate the physical presence of diesel in the soil without having chemical effects. Both vegetable oil and diesel are hydrophobic and have a similar viscosity at room temperature. Thus, they are expected to mix with the soil in a similar way and cause similar physical effects like clogging of pores and reduced water retention or reduced diffusion of oxygen into the soil. However, due to the lack of low molecular substances, vegetable oil is not expected to cause any direct toxic effects on soil microbes and plants. We also hypothesized that the reduced shoot and root biomass and less remediation rate in these soils may have mainly been due to the physical effects of oil. Therefore, the objective of this study was to contribute to a better differentiation between physical and chemical effects of oil via monitoring seedling emergence, plant growth and root biomass.

## Materials and methods

### Soil spiking and stabilization

Soil used in the greenhouse pot experiment was prepared artificially by mixing sand and loess with a ratio of 9:1. Samples taken from this artificial soil mixture were analysed for moisture content (Rondon et al. 2007), soil pH and EC (Quilliam et al. 2013), soil texture, NPK content, total organic carbon (TOC), inorganic carbon and total carbon fraction (Estefan et al. 2013; Gee and Bauder 1986; Rowell 2014). All these parameters were analysed at the start of the experiment (Table 1). After that, artificial soil was spiked with commercial available diesel fuel and edible vegetable oil. The used vegetable oil (normally used for family cooking) was derived from a parent material of sunflower purchased from a supermarket (Hofer) from Tulln, Austria.

**Table 1:** Soil textural class, nutrient status, physico-chemical properties spiking levels and TPH concentrations of soil use for the current experiment

	Parameters	Values	TPH concentration (g/kg DM soil)	
			Initial time (0 day)	Harvesting time (147 days)
Soil texture	Sand (%)	67.20		
	Silt (%)	26.30		
	Clay (%)	6.50		
Soil nutrients (NPK)	Nitrogen (N) (mg kg^−1^)	06.62		
	Phosphorus (P) (mg kg^−1^)	10.73		
	Potassium (K) (mg kg^−1^)	80.46		
Physio-chemical parameters	pH	7.53		
	Electrical conductivity (mS/cm)	143.60		
	Total organic carbon (TOC) %	6.83		
	CaCO_3_ (%)	18.50		
Soil spiking levels	Control (non-contaminated soil)	No oil spiking	0.25 (0.11)	–
	Vegetable oil–spiked soil	2% (*w*/*w*) edible vegetable oil was added	0.26 (0.81)	–
	Diesel oil–spiked soil	2% (*w*/*w*) diesel fuel was added	16.29 (1.03)	9.62 (1.11)

The aim of using vegetable oil was to simulate the physical presence of diesel in the soil without having chemical effects. Both vegetable oil and diesel are hydrophobic and have a similar viscosity at room temperature. Thus, they are expected to mix with the soil in a similar way and cause similar physical effects like clogging of pores and a reduced water retention or reduced diffusion of oxygen into the soil. However, due to the lack of low molecular substances, vegetable oil is not expected to cause any direct toxic effects on soil microbes, or plants. The spiking level was 2% (*w*/*w*) of both oils. These levels of spiking were selected as this concentration is known to have inhibitory effects on germination and plant growth (Cao et al. 2016; Covino et al. 2016; Zhang et al. 2016). The spiked soil was left undisturbed for 4 weeks to achieve equilibration then subsequently filled in plastic pots.

### Greenhouse pot experiment and plant growth conditions

The greenhouse pot experiment was conducted to investigate the chemical and physical effects of diesel fuel and vegetable oil respectively on plant growth and physiological parameters. This experiment was carried out in the greenhouse facility of AIT-BOKU, Tulln, Austria. Plastic pots (2.3 kg) were filled according to the following experimental plan with five replicates for each treatment and arranged in a complete randomized block (CRB) design.

- T1: artificial soil having no amendments (control)
- T2: artificial soil spiked with edible vegetable oil (2% *w*/*w*)
- T3: artificial soil spiked with commercial diesel fuel (2% *w*/*w*)

Subsequently, each pot was sown with a mixture of grass and legume plants by using 240 seeds/pot of Italian ryegrass (IR) and 60 seeds/pot of Birdsfoot trefoil (BT). In the greenhouse, the following conditions (day/night) were maintained for plant growth: light interval 12 h/12 h, temperature 22° C/15° C and relative humidity 50%/60%. The whole experiment was monitored for 147 days. Pots were watered from the bottom when soils seemed to be drying allowing capillary rise for plants. Pots from all the treatments were provided almost the same amount of water keeping in mind the visual observation of pot drying conditions. The levels of manual watering depend on greenhouse temperature and plan biomass, and watering was done in a completely manual way when needed.

### Plant growth and physiological parameters

Seed germination for both IR and BT was observed until 3 weeks after sowing and presented only at the time of the last evaluation. Seed germination (SG) percentage for both plants was calculated separately for each pot. SG (%) was calculated by using formula SG (%) = (Number of germinated seeds/Number of total seeds * 100) as explained by Luo et al. (2017). Parameters like shoot length, fresh and oven-dry biomass and leaf chlorophyll content of both plants were determined after every 49 days. Leaf chlorophyll content was determined with a portable chlorophyll meter (SPAD 502, Minolta, Japan). SPAD values have been used in many studies for approximation of relative chlorophyll contents (Ling et al. 2011; Yang et al. 2014; Monostori et al. 2016). At the time of each cutting, shoot length was kept at 4–5 cm to facilitate rapid sprouting again. In total, we monitored plant growth up to day 147 (3 cuttings). At the end of the experiment, roots were also separated from the rhizosphere and bulk soil and their fresh and oven dry biomass was determined. Exhaustive soil samples were collected from each pot at the end of the experiment with a soil auger/core and stored at − 80 °C until further analysis.

### Soil total petroleum hydrocarbon determination

TPH (C10–C40) concentrations in soil samples collected at the start of the experiment and at the time of final harvesting were determined employing the ISO method 16703:2004 E (Hussain et al. 2018). Three to five grams of soil samples (two sub-samples) was weighted in 40-mL extraction vials which had been pre-sieved to 2 mm. Then, we added 10 mL of acetone (HPLC grade) and 5 mL of extraction solution. One litre of extraction solution comprised n-tetracontane (30 μL/L), n-decane (21 mg/L) and n-heptane (1 L). Subsequently, samples were added to a horizontal shaker (250 rpm) for 60 min. After shaking, distilled water was added and samples were centrifuged with 9000 rpm (SORVALL® centrifuges RC-5C plus). Afterwards, an aliquot of the supernatant was collected by a glass pipette and was transferred into a separate 15-mL glass vial. Collection of the supernatant was repeated twice for each sample. Finally, Florisil (magnesium silicate) was also added to the supernatant for clearing obscures. The transparent extract was transferred to 2-mm GC vials for further chemical analysis. For determining the range of TPHs (C10–C10), the stored sample (2-mL) GC vials were put into an autosampler unit of a gas chromatograph coupled with a flame ionization detector (FID). Analyses were performed using a Hewlett Packard 5890 GC-FID having a 7673 automatic injector. A column having a 30 m*0.25 mm dimension coated with 0.25 μm 5% phenyl 95% methyl polysiloxane stationary phase (DB-5) was used for hydrocarbon analysis. Helium was used as carrier gas. The GC oven was programmed to keep 60 °C for 1 min, followed by 20 °C/min up to 340 °C and then finally 10 min at 340 °C.

### Statistical analysis

Data analyses for all the plant and soil parameters were done using Microsoft Excel and SPSS software packages. The results were calculated and interpreted by analysis of variance (ANOVA), and mean values ± standard deviations were compared between treatments by one-way ANOVA. After testing homogeneity of variance (Levene’s test), Duncan’s test (*p* = 0.05) was used as post hoc test.

## Results

### Seed germination of Italian ryegrass and Birdsfoot trefoil

**Fig. 1:**
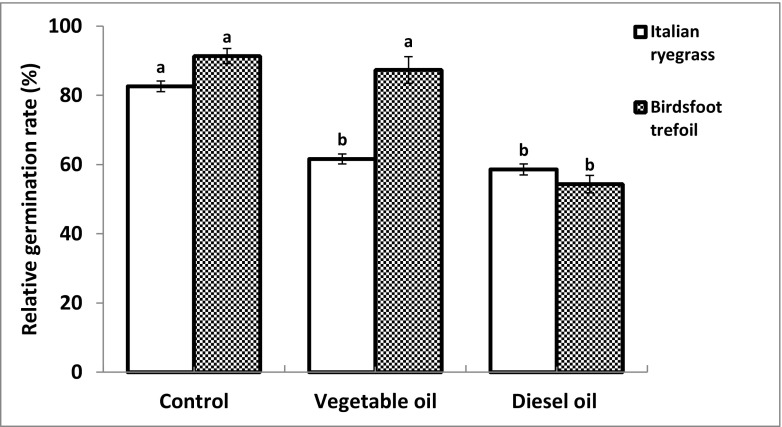
Relative germination rate of Italian ryegrass and Birdsfoot trefoil in non-contaminated soil (control), edible vegetable oil–spiked soil and diesel fuel–spiked soil. Bars represent mean ± SD (*n* = 5). Different letters indicate significant differences between treatments at *p* ≤ 0.05 according to one-way ANOVA followed by Duncan’s test

In the control soil, no significant difference was observed between Italian ryegrass and Birdsfoot trefoil with respect to seed germination (Fig. 1). IR showed the highest seed germination in the control (82.6%), while in vegetable oil and diesel fuel–amended soil, the seed germination was significantly lower with 61.6% and 58.6% respectively. The germination rate of BT was highest in the control soil (91.3%) with no significant decrease in soil spiked with vegetable oil (87.3%) but a significant decrease in the diesel fuel–amended soil (54.3%).

### Plant shoot biomass

**Fig. 2:**
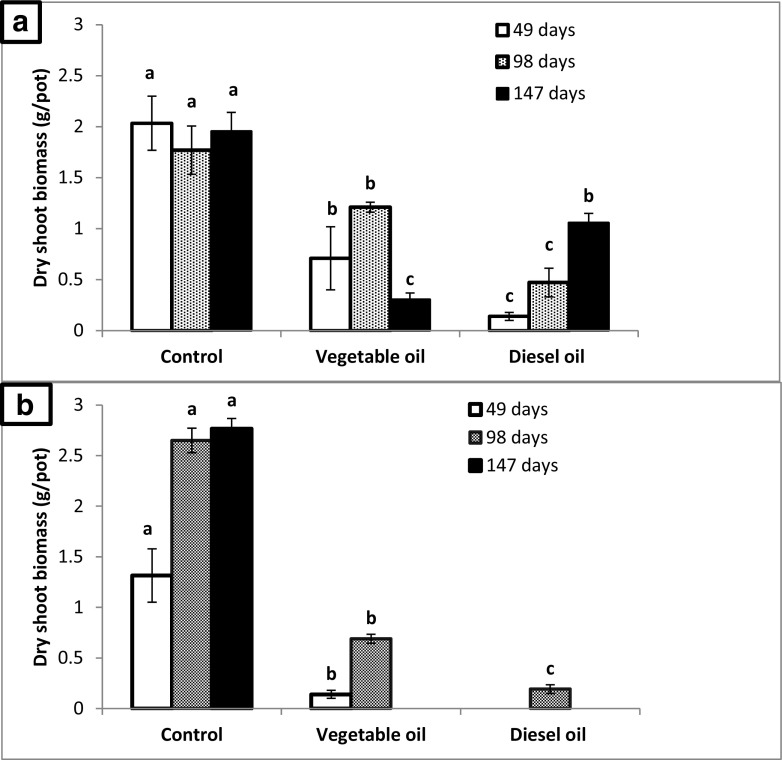
Shoot biomass (g/pot) at three cutting times of **a** Birdsfoot trefoil and **b** Italian ryegrass. Bars show means and standard deviations. Different letters indicate significant difference between three treatments at each harvest (Duncan’s test, *p* ≤ 0.05)

Italian ryegrass showed a distinctly higher production of shoot biomass in the control soil as compared to vegetable oil and diesel fuel–amended soil during all three cutting intervals (Fig. 2b). After 49 days, Italian ryegrass had a significantly lower shoot biomass in soil spiked with vegetable oil, whereas no plant could be harvested from soils spiked with diesel fuel. After 98 days, aboveground biomass decreased significantly from control vial vegetable-spiked soil to diesel fuel–spiked soil. After 147 days, IR showed harvestable shoot biomass only in the non-spiked control soil. No shoot biomass was recorded as IR failed to sprout again after the second cutting in both oil-amended soils.

The legume plant Birdsfoot trefoil showed a similar pattern in the first two cutting intervals (49, 98 days). A significantly higher shoot biomass for BT was recorded in control soils as compared to oil-amended soils. Moreover, BT grown on diesel fuel–amended soil showed a significantly reduced shoot biomass compared to BT grown on soil spiked with vegetable oil (Fig. 2a). At the time of the final harvest (147 days), however, the diesel fuel–amended soil led to a statistically higher shoot biomass compared to vegetable oil–amended soil, while control plants had the significantly highest biomass (Table 2).

**Table 2:** SPAD values of Italian ryegrass and Birdsfoot trefoil influenced by vegetable oil and diesel fuel and non-contaminated soil. SPAD values were recorded at 49, 98 and 147 days after sowing. Different lowercase letters among different treatment show significant difference by one-way ANOVA fallowed by Duncan’s test (*p* ≤ 0.05)

	Chlorophyll content of leaves (SPAD values)					
	Italian ryegrass			Birdsfoot trefoil		
	49 days	98 days	147 days	49 days	98 days	147 days
Control soil	25.4 ± 1.6	34.7 ± 2.2a	22.9	40.8 ± 1.3a	35.3 ± 2.9a	37.4 ± 2.1a
Vegetable oil–amended soil	NM*	19.5 ± 0.7b	NM	12.0 ± 2.0c	22.5 ± 2.7b	14.1 ± 2.3c
Diesel fuel–amended soil	NM	08.7 ± 1.8c	NM	17.0 ± 0.7b	17.6 ± 1.2c	21.0 ± 1.6b

NM means non-measureable value because IR leaves were not expanded

**Fig. 3:**
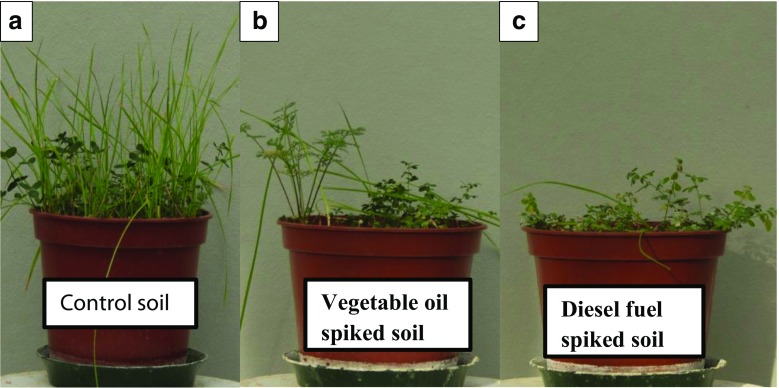
Representative photos of *Italian ryegrass* and *Birdsfoot trefoil* at the time of first cutting (49 days) in **a** control non-contaminated soil, **b** soil spiked with edible vegetable oil (2% *w*/*w*) and **c** soil spiked with commercial available diesel fuel (2% *w*/*w*)

During the whole course of the experiment, a distinct species shift was recorded (from mixed plants to monoculture of BT in oil-amended soils especially after the first cutting), while the control showed a uniform mixture of grass and legume plants. Plants grown in oil-amended soil produced significantly less shoot biomass compared to plants grown in non-contaminated control soil (Fig. 3). In terms of cumulative shoot biomass, no statistically significant difference was observed between vegetable oil and diesel fuel–spiked soils (Table 4). During the whole course of the experiment (at the time of all harvests), shoot lengths of IR and BT were severely affected by the presence of oils as compared to non-amended soil. Within vegetable oil and diesel fuel treatments, no significant differences were observed (Table 3).

**Table 3:** Length of Italian ryegrass and Birdsfoot trefoil influenced by vegetable oil and diesel fuel presence and non-contaminated soil. Shoot length was recorded at 49, 98 and 147 days after sowing. Different lowercase letters show significant difference among treatments (one-way ANOVA followed by Duncan’s test as post hoc test (*p* ≤ 0.05)

	Shoot length (cm)					
	Italian ryegrass			Birdsfoot trefoil		
	49 days	98 days	147 days	49 days	98 days	147 days
Control	19 ± 3.5a	22.0 ± 2.5a	13 ± 4.0	10.2 ± 2.2a	13.4 ± 3.3a	15.0 ± 4.4a
Vegetable oil–amended soil	2.5 ± 1.5b	10.0 ± 1.5b	NA	3.1 ± 2.2b	8.0 ± 2.2b	7.0 ± 2.5b
Diesel fuel–amended soil	3.0 ± 1.7b	9.0 ± 2.4b	NA	5.6 ± 2.7b	7.0 ± 2.2b	5.4 ± 1.5b

### Root biomass

The highest total root biomass was recorded for plants grown in the non-amendment control soil followed by a significantly lower value in diesel oil–spiked soil and again a significantly lower value in soil amended with vegetable oil (Table 4).

**Table 4:** Shoot biomass (g/pot) of both plants recorded at three different cutting intervals, root biomass (g/pot) recorded at the time of last cutting interval (147 days), root/shoot ratio and cumulative aboveground biomass of both studied plants. Different lowercase letters behind numbers show significant difference among treatments (one-way ANOVA followed by Duncan’s test as post hoc test (*p* ≤ 0.05)

	Shoot biomass (g/pot) Cumulative for both plants			Root biomass (g/pot)	Root/Shoot ratio	Cumulative above ground biomass for three cuttings
Treatments	49 days	98 days	147 days	147 days	Ratio	49 days + 98 days + 147 days
Control	3.35 ± 0.53a	4.42 ± 0.36a	4.74 ± 0.28a	4.57 ± 0.25a	0.97 ± 0.05b	12.51 ± 0.50a
Vegetable oil–amended soil	0.85 ± 0.35b	1.90 ± 0.09b	0.31 ± 0.08c	0.31 ± 0.11c	1.07 ± 0.48b	3.06 ± 0.60b
Diesel fuel–amended soil	0.14 ± 0.04c	0.66 ± 0.18c	1.05 ± 0.18b	1.67 ± 0.15b	1.62 ± 0.22a	1.85 ± 0.44b

### SPAD values

The SPAD values of Italian ryegrass generally showed the highest values in the control compared to plants grown in oil-amended soil (Table 2). At the time of the first and the third cutting, SPAD values were only measurable for plants in the control soil due to a poor expansion of IR leaves in oil-amended soils. At the time of the second cutting, chlorophyll content was highest in plants grown on control soil, significantly less in plants grown on soil amended with vegetable oil and again significantly less in plants grown on diesel-spiked soil. Birdsfoot trefoil showed the same trend in chlorophyll content at the time of the first and the third cutting while it was different at the second cutting interval. At 49 and 147 days, BT grown on vegetable-amended soils showed the lowest chlorophyll content as compared to diesel oil–amended soil and again a significant higher value was reported for the non-contaminated control. However, at day 98, SPAD values of BT were significantly more reduced by diesel oil amendment as compared to vegetable oil addition.

### TPH degradation by the action of legume and grass plants

Analysis of hydrocarbons was performed in all the variants, but the removal percentage was only calculated for diesel-spiked soil due to negligible concentrations of hydrocarbons in non-contaminated and vegetable oil–spiked soil. The removal percentage was calculated by a formula as explained in Hussain et al. (2018): 100 × [(*C*_i_ − *C*_f_)/*C*_i_], where *C*_i_ stands for the TPH concentration at the initial stage (at the time of seed planting and after soil stabilization) and C_*f*_ shows the TPH concentration at the harvesting stage (after 147 days) of experiment. While comparing the TPH concentrations at two mentioned time slots, a substantial decrease (more than 40%) was recorded at the harvesting time.

## Discussion

Within the scope of phytoremediation, rhizoremediation is gaining enormous attention (Gerhardt et al. 2017) and has been accepted as a green or sustainable solution for the remediation of organic contaminants (Song et al. 2016; Dubey and Fulekar 2013) especially petroleum and its derived products (Hou et al. 2015). Studies on different influencing factors are important for a better understanding of plant growth in TPH-impacted soils during rhizoremediation. Various sensitivities of plant species to different types of contaminants have been reported (Baker 1970; Chaîneau et al. 1997; Gauvrit and Cabanne 1993). In this experiment, we tried to separate physical effects from chemical effects by comparing the effects of soil spiked with vegetable oil (expected to exert only physical effects) with soil spiked with diesel fuel (expecting to exert both physical effects and chemical toxicity). The aim of using vegetable oil was to simulate the physical presence of diesel in the soil without having chemical effects. Both vegetable oil and diesel are hydrophobic and have a similar viscosity at room temperature. Thus, they are expected to mix with the soil in a similar way and cause similar physical effects like clogging of pores and a reduced water retention or reduced diffusion of oxygen into the soil. However, due to the lack of low molecular substances, vegetable oil is not expected to cause any direct toxic effects on soil microbes, or plants.

While the germination rate of Italian ryegrass was significantly affected by both oil amendments, Birdsfoot trefoil only showed a significant reduced germination rate in soil spiked with diesel fuel. Thus, the physical effects of oil on soil properties appeared to have no significant impact on seed germination of BT in our experiment. The increase in soil hydrophobicity and consequently a reduced water potential by the addition of vegetable oil may have been responsible for the reduced seed germination of IT in the vegetable oil–amended soil. Different requirements of grass seeds and legume seeds in respect to water supply during germination might thus explain the different sensitivity in germination of these two species (Merkl et al. 2005; McWilliam et al. 1970). Additionally, the formation of surface slicks on the seed surface might have reduced uptake of oxygen and water by seeds resulting in poor germination. Chances of making oily slicks on seed surface of IR (due to its roughness) are more as compared to BT (smooth seed surface). This might correspond to the low germination of IR compared to BT in vegetable oil–amended soil. Diesel fuel amendment reduced the seed germination of both species as compared to the untreated control soil. This reduction in seed germination might be explained by the physical and chemical effects posed by diesel oil addition. This fact is well established in literature that different constituents of diesel fuel (alkanes, aromatics and polycyclic aromatic hydrocarbons) are toxic to many tested plant species (Chaîneau et al. 1997; Adam and Duncan 2002; Huang et al. 2004; MacKinnon and Duncan 2013; Macoustra et al. (2015); Panchenko et al. 2016). It appears that reduced germination of Birdsfoot trefoil is mainly due to chemical toxicity of diesel fuel, while reduced germination of Italian ryegrass seeds was affected mainly by physical effects caused by oil contaminants in soil. Apart from germination, plant growth is an important parameter that is known to be reduced in oil-spiked soil (Marín-García et al. 2016; Gartler et al. 2014; Gong et al. 2008; Li et al. 1997). Reduction of plant biomass in diesel-impacted soils has already been studied and may be attributed to the combination of physical and chemical effects created by the presence of diesel oil. Some reports linked the reduction of plant biomass to adverse soil conditions caused by hydrocarbons (Lacalle et al. 2018; Langer et al. 2010) while others explained it with the direct toxic effects of diesel and its constituents to plants itself (Bell et al. 2014; Basumatary et al. 2012). Diesel oil in soil produces adverse conditions such as higher acidity, lower availability of essential nutrients (C, N and P), reduced exchangeable cations and lower microbial activity (Al-Asheh et al. 1999; Siddiqui and Adams 2002). The reduction in plant biomass in the presence of diesel fuel has been reported in many studies (Cui et al. 2016; Han et al. 2016; Shahzad et al. 2016; Xun et al. 2015). We investigated plant growth by cutting the plants at three times to differentiate between physical and chemical effects of oil spiked soil on plant growth. In the current study, severe growth impairment (stunted growth) was observed in both vegetable oil– and diesel fuel–amended soil as compared to the non-contaminated control in both plant species. However, legume and grass plants showed different responses throughout the growth period. While, up to the second cutting both plants showed a higher shoot biomass in vegetable oil compared to diesel oil, at the 3rd cutting, higher shoot biomass was recorded for BT in the diesel-spiked soil as compared to the vegetable oil–amended soil. Different mechanisms and processes may be responsible for the reduced plant growth in oil-spiked soil. Physical effects refer to the lower water potential, faster drying (as visible by earlier appearance of lighter colours of the soil surface), reduced oxygen availability and lower nutrient availability, while chemical effects may be caused by toxic low molecular weight components of diesel as well as nutrient imbalances due to enhanced availability of carbon. In some studies, water repellence and low matrix potential were made responsible for reduced shoot biomass in oil-impacted soils (Gartler et al. 2014; Li et al. 1997; Marín-García et al. 2016). Some other researchers linked the lower plant growth to insufficient aeration due to oil-impacted soils (Adenipekun et al. 2008; Odjegba and Sadiq 2002; Smit et al. 1989). Such stress factors can lead to responses of plants at the physiological and biochemical levels (Ma et al. 2013; Saraeian et al. 2015; Zhang et al. 2015). These include effects on molecules indicating enhanced oxidative stress like (malondialdehyde, hydrogen peroxide, chlorophyll, proline and glutathione in its reduced and oxidised form) as well as enzymes of the antioxidative apparatus (superoxide, dismutase, catalase, ascorbate and peroxidase) (Zhang et al. 2015). In our study, the physical effects of vegetable oil alone appeared to have a strong inhibitory effect on the growth of Italian ryegrass and Birdsfoot trefoil, whereas the latter appeared to be less sensitive. Additional chemical effects due to toxic low molecular compounds in diesel oil further reduced plant growth of both species up to the third harvest for IR and the second harvest for BT, while at the third harvest BT showed more biomass in diesel fuel–spiked soil compared to soil spiked with vegetable oil. Moreover, the results of the current study indicate the respective plant sensitivity in a mixed cropping pattern. In some studies, legumes were more sensitive as compared to grasses toward hydrocarbon stress (Banks et al. 2003; Gartler et al. 2014). However, in the current investigation, the legume plant (BT) showed more pronounced biomass as compared to the grass plant (IR). Recent studies (Liu et al. 2017; Lv et al. 2014) observed similar findings of legume success over grasses in a mixed cropping pattern.

The reduction in chlorophyll content corresponded quite well with the reduction in biomass. Accordingly, diesel fuel had a stronger effect on IR as compared to BT. The chlorophyll content can be regarded as a sensitive bio-indicator for stress factors since these lead to an over-reduction of the thylakoid membrane in chloroplasts that can culminate in photooxidation of chlorophyll and other components of the electron transport chain (Cui et al. 2016; Han et al. 2016; Zhang et al. 2012). Lower chlorophyll contents in diesel-impacted soils have already been reported in many documents and attributed to the direct toxic effect of diesel fuel exerted on plants (Achuba 2006; Achuba and Okoh 2014; Han et al. 2016; Lin et al. 2008) and alteration of chemical properties of soils (Balasubramaniyam and Harvey 2014, 2015). Diesel fuel contamination in soils alters the nutritional status of soils especially availability of nitrogen and phosphorus (Arslan et al. 2014b; Choi and Chang 2009; Nie et al. 2011; Tahseen et al. 2016) which also can cause a decrease in chlorophyll content of leaves. Our study showed comparable results with other studies (Wang et al. 2005, 2008) in which reduced chlorophyll contents (Chl a, Chl b and Chl total) of rice and reed plant were recorded in the presence of petroleum contamination. The unique capabilities of legume plant (BT) to fix atmospheric nitrogen (as supported by observation of nodule formation on root surface) cope with different environmental stresses and enhanced nutrient acquisition (Escaray et al. 2012; Lee et al. 2008; Merkl et al. 2005; Panchenko et al. 2016) may explain the higher photosynthetic performance in all three cutting intervals as compared to grass (IR).

### Effects on root biomass

Roots are considered as ecological drivers of hydrocarbon rhizoremediation by producing root exudates and hormones that can provide a favourable micro-environment for the growth of microbes and influence plant development (Rohrbacher and St-Arnaud 2016). It can occur as roots can provide microbial substrate to the soils to enhance the growth of hydrocarbon-degrading microbes. Roots can also stimulate oxidative degradation of TPH by improving aeration and can widen the scope of rhizoremediation for a “trapped” fraction of contaminants to become accessible for degrader bacteria (Leigh et al. 2002). Additionally, root turnover is supposed to be an important contributor in improving soil aeration by creating air channels by the process of root death and decay (Agostini et al. 2013; Greenwood et al. 1982; York et al. 2016). Results from the current study support the argument that root development was strongly affected by the presence of both vegetable oil and diesel fuel. Both oils showed lethal and drastic effects on root biomass of studied plants as compared to the control.

Plants grown in diesel fuel–spiked soil had a higher root biomass compared to plants grown in vegetable oil–spiked soil. This seems to be linked to the higher shoot biomass of BT at the last cutting which was connected to the observed species shift. A similar species shift favouring the growth of BT was observed with vegetable oil. Thus, it appears that the legume BT could better handle the physical stress factors caused by the oil like reduced soil water content, lowered oxygen supply and as a potential consequence lower nutrient availability. Poor soil structure (i.e. the size of particle aggregates and the overall soil porosity) resultant by the addition of vegetable oil may also be responsible for reduced root development. High water repellence creates compacted soils (high soil strength), a physical stress, which may also influence root elongation (Gregory 2008; Gregory et al. 2009). These current findings are similar to those of Hopkins et al. (1950), where authors reported that root biomass was highly affected by the availability of oxygen. Additionally, a linkage between nutrient accumulation, their transport into the root zone and oxygen supply was established. Interestingly, at the third harvest, the aboveground biomass of BT was significantly higher in diesel oil–spiked soil compared to vegetable oil–spiked soil. This corresponds quite well with the higher root biomass at this time. While in the control no shift in the abundance of both BT and IR could be observed, the growth of the grass (IR) was affected more strongly by both vegetable oil and diesel oil, leading to a predominance of the legume (BT). The better growth of BT was more pronounced in diesel oil–spiked soil and was correlated with a higher root biomass. Nevertheless, diesel-impacted soils showed significant negative effects on root biomass as compared to the non-contaminated control. Our findings are in accordance with other findings (Euliss et al. 2008; Nie et al. 2011; Peng et al. 2009; SHABIR et al. 2016; Shirdam et al. 2008; Tang et al. 2010) who observed reduced root biomass in the presence of diesel oil.

Our results show that there are differences among species in sensitivity to physical effects caused by oil contamination of soil. Germination and growth of Italian ryegrass were strongly affected already by physical effects caused by vegetable oil, while the legume Birdsfoot trefoil showed much less effects that could be attributed to physical effects caused by oil in the soil.

### Implication for rhizoremediation studies

Planting IR and BT in mixture showed a 40% reduction in TPH contents of diesel-impacted soils. The favourable remediation abilities of these two plants have been studied extensively for diesel-impacted soils. The mechanism responsible for TPH dissipation is known as rhizoremediation—where joint action of plants and their associated microorganism can degrade petroleum hydrocarbons in the active zone of the rhizosphere (Hussain et al. 2018). The current dissipation of hydrocarbons can be explained by the stimulation of rhizospheric effects supported by roots of legume and grass plants. Additionally, plants can also provide exudates, enzymes and oxygen for microbial communities that also help in dissipation of TPH (Martin et al. 2014). The results of the current study are comparable with many other reports like Kaimi et al. (2007), Andria et al. (2009)), Yousaf et al. (2010), Arslan et al. (2014), Afzal (2010) and Ikeura et al. (2016) in which authors showed enhanced dissipation of TPH by the action of grass or legume plants. The current findings have given insights for successful rhizoremediation trials elaborating the reasons for the reduced shoot and root biomass by the action of physical or chemical or combined effects of oil’s presence in soils. The study also extends systematic investigations of chlorophyll contents for both plants affected by vegetable oil and diesel fuel. We concluded that during rhizoremediation/phytoremediation trials, not only does chemical toxicity of contaminants play a vital role in plant growth developments but also that physical effects may influence the overall success. The physical effects described by the reduced oxygen levels, nutrient accessibility and poor structured soils may also explain the lesser success in aged-contaminated soils up to a limited extent. The physical effects caused by oil contaminations of soil are thus expected to be a major factor causing reduced plant growth in aged-contaminated soils. Nevertheless, to assess the full picture of the physical effects of oil’s presence in rhizoremediation studies, we recommend extending the scope of this study to soil microbiota in relation to changes observed in soil properties. Also, the extent of the current study can be widened via performing a detailed investigation of plant physiological measurement by analysing hydrogen peroxide (H_2_O_2_), monodehydroascorbate (MDA), superoxide dismutase (SOD), catalase (CAT), ascorbate peroxidase (APX), phenols, proline and several other antioxidants.
